# Spherical PEG/SiO_2_ promising agents for Lamivudine antiviral drug delivery, a molecular dynamics simulation study

**DOI:** 10.1038/s41598-023-30493-3

**Published:** 2023-02-27

**Authors:** Sahar Razzaghi, Mohsen Vafaee, Bahar Kharazian, Mokhtar Nasrollahpour

**Affiliations:** 1 Department of Chemistry, Faculty of Science, Tarbiat Modares University, P.O. Box 14115-175, Tehran, Iran; 2 Faculty of Pharmacy, Nanotechnology Research Center, Tehran University of Medical Sciences, P.O. Box 14155-6451, Tehran, Iran

**Keywords:** Physical chemistry, Theoretical chemistry, Computational biophysics

## Abstract

Spherical nanocarriers can lead to a bright future to lessen problems of virus infected people. Spherical polyethylene glycol (PEG) and spherical silica (SiO_2_) are novel attractive nanocarriers as drug delivery agents, especially they are recently noticed to be reliable for antiviral drugs like anti-HIV, anti-covid-19, etc. Lamivudine (3TC) is used as a first line drug for antiviral therapy and the atomic view of 3TC-PEG/SiO_2_ complexes enable scientist to help improve treatment of patients with viral diseases. This study investigates the interactions of 3TC with Spherical PEG/SiO_2_, using molecular dynamics simulations. The mechanism of adsorption, the stability of systems and the drug concentration effect are evaluated by analyzing the root mean square deviation, the solvent accessible surface area, the radius of gyration, the number of hydrogen bonds, the radial distribution function, and Van der Waals energy. Analyzed data show that the compression of 3TC is less on PEG and so the stability is higher than SiO_2_; the position and intensity of the RDF peaks approve this stronger binding of 3TC to PEG as well. Our studies show that PEG and also SiO_2_ are suitable for loading high drug concentrations and maintaining their stability; therefore, spherical PEG/SiO_2_ can reduce drug dosage efficiently.

## Introduction

Acquired immunodeficiency syndrome (AIDS) is one of the most infectious diseases in the whole world. It ranks among the top four causes of death throughout the globe, particularly in Africa. It was discovered in the early 1980s that the Human immunodeficiency virus (HIV) is the causative agent of AIDS; According to the UNAIDS latest available data in 2021 about 84.2 million people worldwide have been infected since the start of the epidemic. Almost 38.4 million people are currently living with this pandemic, where women account for 54% of the population while Children (age 0–14) constituted 1.7 million of the total people living with HIV^1,2^.

One of the best ways to reduce the brunt of viral illnesses is using drugs. Since the discovery of AIDS in 1981 and its cause, the HIV virus, in 1983, dozens of new antiretroviral medicines to treat HIV have been developed. Antiviral therapy stops HIV from multiplying and can suppress HIV to undetectable levels in the blood. This allows a person’s immune system to overcome infections and prevent the progression of AIDS. Currently, antiviral therapy is available for the control of HIV but like other drugs, it can have serious side effects. Researchers throughout the world are trying to explore and develop more reliable and safe drugs from natural resources to manage HIV infection. A wide range of medicinal plants have been studied and reported to have potential against HIV but the exact mechanism of action is still not known and it is just clear that they have an impact to interrupt the life cycle of HIV. Although medicinal plants have the potential for the management of HIV/AIDS, more studies are needed to reveal efficacy and safety concerns by conducting clinical trials at a vast level to explore the therapeutic impact of them. Lack of sufficient bright data for medicinal plants makes people use chemical-based drugs more in treating HIV^3,4^. Antiviral drugs mainly inhibit viral infection and cell proliferation, reducing the disease’s intention. The most used drugs in the medical therapy of viral sicknesses are Lamivudine, Zidovudine, Telbivudine, Stavudine, Adfovir, Infoviride, Indinavir, Acyclovir, Foscarnet, and Tenofovir^5^.

According to the literature, the initial treatment regimen for adults and adolescents with HIV generally consists of nucleoside reverse transcriptase inhibitors (NRTIs), usually lamivudine (3TC) is used as first line treatment drug for these patients. Fixed doses and once-daily regimens are preferred^6^. Amino-1-[(2R, 5S)-2-(hydroxymethyl)-4, 1, 3-oxathiolan-5-yl]-1, 2-dihydropyrimidin-2-one (Lamivudine) is a water-soluble potent nucleoside analog reverse transcriptase inhibitor^7^. Lamivudine has various side effects, such as thrombocytopenia, paresthesia, anorexia, abdominal cramps, depressive disorders, and skin rashes, depending on the dosage used. Therefore, it is clear that reducing the intake dose is a way to reduce the side effects and toxicity of the drug^8^. Lamivudine competitively inhibits the activity of the reverse transcriptase enzyme, which acts as an inhibitor of DNA synthesis. Targeting the drug by nanoparticles (NPs) is expected to be beneficial in reducing the used dose of a drug, reducing side effects, and maintaining drug delivery to the target organ are the main advantages of using NPs^9,10^.

NPs such as magnetic^11^, lipid-based^12^, polymeric^13^, dendrimers^14^, nanotubes, nanowires, etc. are mainly used as carriers for delivering the drug to the targeted area and protecting them from degradation. Magnetite (Fe_3_O_4_) is one of the most common NPs in biomedicine. Their main advantage over other carriers is their easy synthesis and reaction to the magnetic field, so the movement of drug-loaded NPs for targeted drug delivery can be controlled by applying external magnetic force^15^. As magnetite NPs (MNPs) enter the biological environments, they usually tend to aggregate. The key is creating inert coatings such as silica or PEGylating on the surface of the MNPs^16^. Coatings have got lots of advantages, such as improving the dispensability and colloidal stability of carriers, protecting their surface from oxidation, providing a surface for conjugation to the drug molecules, increasing circulation time by avoiding clearance by the reticuloendothelial system, increasing biocompatibility, and reducing nonspecific interactions^4^. PEG has been widely utilized in biomedical applications such as bioconjugation, drug delivery, biosensing, imaging, and tissue engineering. PEG is either directly conjugated with drugs or attached to the surface of drug-encapsulating nanomaterials (a technique known as PEGylation) to augment in vivo stability and solubility and reduce clearance rate from blood circulation, thus optimizing drug efficacy. PEGylated NPs become hydrophilic and can lead to NPs with a significantly improved circulation lifetime compared to unmodified NPs. Additionally, the flexible hydrophilic PEG chains could allow PEGylated NPs to quickly diffuse through mucin fibers for the effective local release of drugs^17,18^.

Shahabadi et al. studied the superparamagnetic NPs coated with silica to deliver Lamivudine and DNA to AGS and MCF-7 cancer cell lines. According to their results, this nanocarrier can double the drug’s effect^19^. Wang et al. investigated the mechanical properties of Polyethylene terephthalate (PET)/silica composites using MD simulations. The simulation results indicate that nanocomposites have higher mechanical properties than composites in pure PET systems, confirming a stronger interaction between the polymer and silica^20^.

The spherical shape of silica coating has a larger surface area and less toxicity, and its good thermal and mechanical properties can improve the properties of composites^21^. Match et al. investigated the adsorption of ibuprofen on various forms of silica NPs using atomic models. Their results show that the adsorption mechanism is based on hydrogen bonding and the pores on these NPs affect the drug’s loading, dispersing, and kinetics^22^. Also, Bardi et al. showed that silica NPs have a honeycomb-like structure with hollow channels that provide a high surface area (> 900 m^2^/g) that can absorb a large amount of biologically active molecules^23^.

For the first time, molecular dynamic simulations is utilized to investigate the reliability of spherical polyethylene glycol (PEG) and spherical silica (SiO_2_) for delivering drugs. Spherical PEG/SiO_2_ are novel attractive nanocarriers proposed as drug delivery agents. It is recently explained that both carriers show less toxicity in spherical shapes rather than triangles, cylinders, and other shapes^24^. Also they are lately noticed to be reliable for antiviral drugs like anti-HIV, anti-covid-19, etc. in experimental studies^6^. Atomic view of 3TC-PEG/SiO_2_ complexes enable scientist to help improve the treatment of patients with viral diseases.

Based on the mentioned facts, the interaction of 3TC molecules with SiO_2_ and PEG-coated MNPs is investigated in this work using MD simulation. In this regard, the adsorption mechanism of 3TC on both carriers and the effect of drug concentration on the loading process are evaluated. The stability of the system was evaluated using the root mean square deviation (RMSD) in addition to the solvent accessible surface area (SASA) of 3TC in the system.

## Materials and methods

Molecular dynamics (MD) simulation, as a helpful tool, give microscopic insights into the system^25^. All simulations were performed using the NAMD package^26^ to investigate the adsorption of 3TC on spherical PEG and SiO_2_ carriers and analyze the stability of complexes. NAMD enable high-performance classical simulation of biomolecules in realistic environments. It is capable to be performed on GPU processors, and is easy to perform for large number of atoms in an all atom simulation. CHARMM27 is used for this study because it was efficiently utilized in previous studies on biomolecules^9^. It contains all parameters for our systems (carrier and drug) and it is complete for our complexes^27,28^.

The structures were visualized by Visual Molecular Dynamics (VMD)^29^. The TIP3P water model was used for all simulations^30^. To determine non-bonded interactions, 6–12 Lennard–Jones potential energy was utilized^31^. All systems were simulated with a time step of 1 femtosecond under the isothermal-isobaric ensemble (NPT). The temperature was kept at 310 K during the simulation using a Nose–Hoover thermostat^32^ and a pressure of 1 bar. The cut-off distance for Van der Waals and electrostatic interactions was 10 Å^33^.

**Figure 1:**
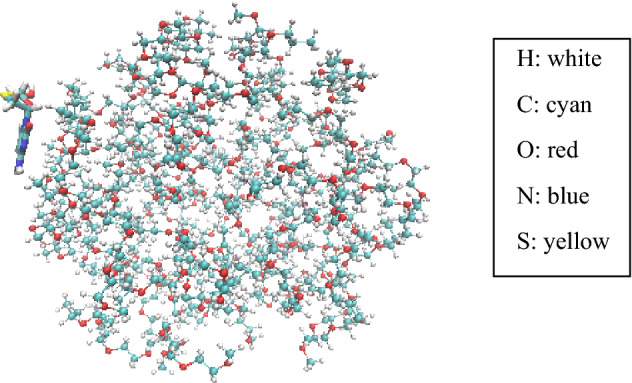
Initial configurations of 3TC on PEG (water box and ions are omitted for better view).

**Figure 2:**
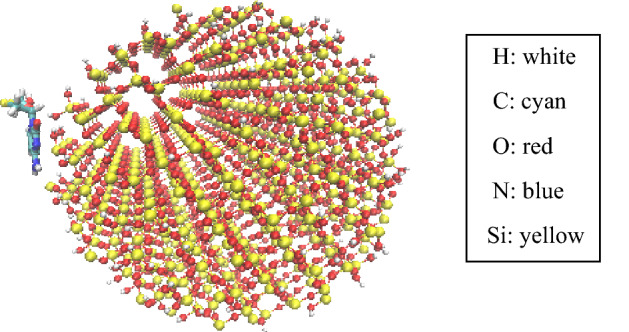
Initial configurations of 3TC on SiO_2_ (water box and ions are omitted for better view).

The SiO_2_ and PEG were kept fixed in this study to accelerate computation. SiO_2_ sphere with a 30 Å radius was made using the Material Studio package. PEG with 40 monomers was formed in globular orientation using PACKMOL^34^. The initial configurations of drug-carrier systems are shown in Figs. 1 and 2. The crystal structure of 3TC is obtained from Protein Data Bank (ID: DB00709). The drug was positioned at 1.06 nm above SiO_2_ and PEG surface. Sodium (Na^+^) and chloride (Cl^−^) ions were added to both systems to ensure that the system was neutralized.

## Results and discussion

MD simulations are used to investigate the atomic scale of 3TC adsorption on various carriers. Different driving forces such as covalent bonding, non-bonded interactions, and hydrogen bonding control the adsorption phenomena of multiple species on other carriers revealed by MD simulations^35^. In this regard, a molecular-scale understanding of nanocarrier loading by drug molecules is necessary to develop the best carrier-drug complexes.

In the present work, by using the MD simulations, we investigate the adsorption of 3TC molecules on SiO_2_ and PEG nanocarriers. The stability of the systems, the radius of gyration (Rg), radial distribution function (RDF), number of hydrogen bonds in systems, SASA, and non-bonding energy of systems are studied. According to the results, the RMSD of 3TC on PEG shows less fluctuation compared to the SiO_2_ carrier. Also, the RDF values for the drug in the vicinity of two carriers indicate that the drug loading on the PEG carrier is better than SiO_2,_ which is confirmed by more hydrogen bonding between 3TC and PEG carrier. Also, SASA calculations show that the availability of solvent for 3TC on PEG is less than SiO_2_ carrier which arises from more interactions between 3TC and PEG. In brief, according to the simulation results, PEG is a better carrier than SiO_2_ for antiviral 3TC to help HIV-infected patients.

### Root mean square deviation (RMSD)

The root mean square displacement (RMSD) measures the mean distance of atoms and gives a steady value that proves the system has attained equilibrium conditions (Eq. 1)^36^

1 $$RMSD=\sqrt{\frac{1}{N}\sum \limits_{i=1}^{N}{d}_{i}^{2}},$$

where *N* is the number of atoms, and ${d}_{i}$ is the distance between the *i*th pair of corresponding atoms^37^.

**Figure 3:**
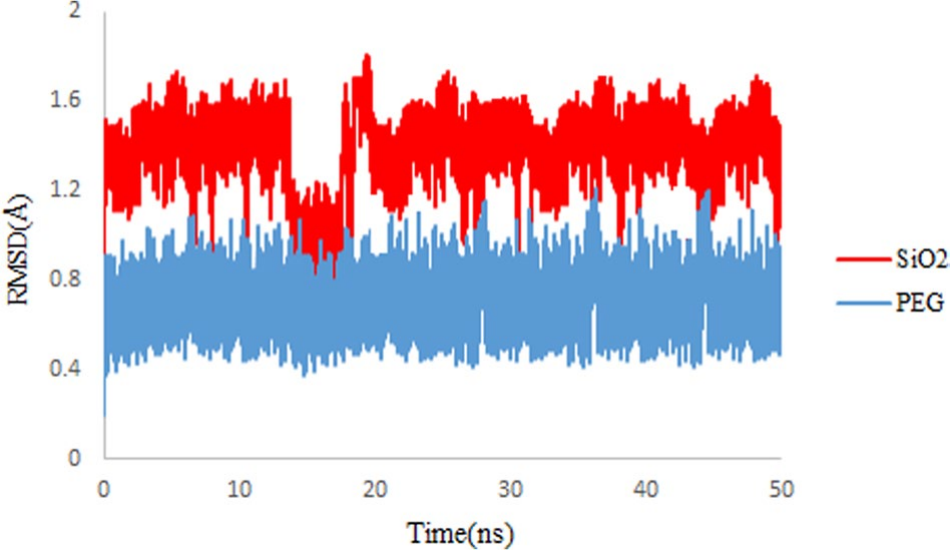
RMSD values of 3TC on SiO_2_ (red) and 3TC on PEG (blue) complexes during 50 ns simulation.

Figure 3 illustrates the RMSD values for drug molecules in simulated systems. For both simulated systems, the convergences of RMSD values for drug molecules confirm the equilibrium of the 3TC on SiO_2_ and 3TC on PEG complexes. Also, it can be seen that convergence for 3TC on PEG is reached faster, and it demonstrates less noise than the RMSD values for 3TC on SiO_2_. Results show that 3TC molecules adsorbed on PEG have stronger non-bonded interactions.

### Solvent accessible surface area (SASA)

**Figure 4:**
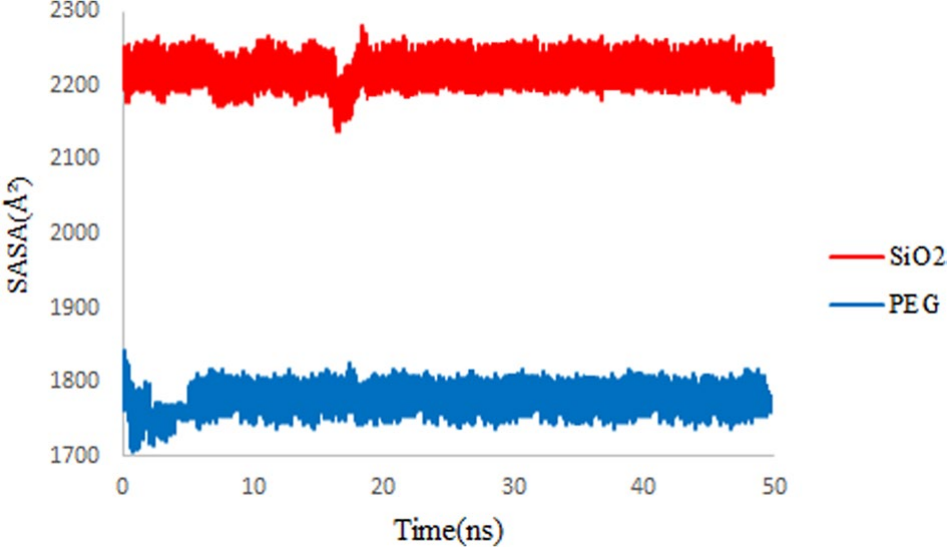
SASA values of 3TC on SiO_2_ (red) and 3TC on PEG (blue) complexes during 50 ns simulation.

SASA is prescribed as the structural access to the solvent that envelopes the outside of a molecule. SASA also shows structural packaging, so a system with high molecular packing will display a low SASA^38^. The SASA values are calculated employing Eq. (2).

2 $$SASA=\sum \left[\frac{R}{\sqrt{{R}^{2}-{Z}_{i}^{2}}}\right]\cdot {L}_{i}\cdot D , D=\frac{\Delta Z}{2}+{\Delta }^{^{\prime}}Z,$$

where ${L}_{i}$ is the length of an arc drawn on a given section $i$; ${Z}_{i}$ and $\Delta Z$ are the perpendicular distance from the center of the sphere to section *i* and the spacing between sections, respectively. $\Delta^{\prime}Z$ is $\frac{\Delta Z}{2}$ or $R-{Z}_{i}$, whichever is smaller^6^. Figure 4 represents the calculated SASA plots for both simulation systems. As shown, the SASA values for the 3TC-PEG complex are converged to lower values rather than the 3TC-SiO_2_ complex. The greater SASA values of the 3TC-SiO_2_ complex in comparison with the 3TC-PEG complex prove that the adsorption of 3TC on PEG leads to a more compact system that agrees with RMSD results.

### Radius of gyration (Rg)

**Figure 5:**
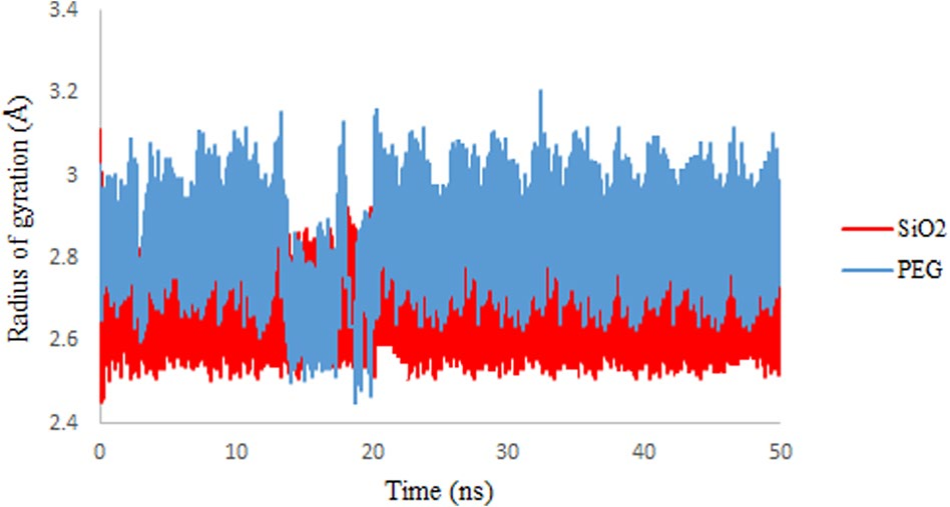
Rg values of 3TC adsorbed on SiO_2_ (red) and PEG (blue) during the simulation time.

The Rg is a measure of the elastic stability of a section. Depending on which axis is considered, a section has several values of rotation radius. To illustrate systems dimensions and measure the position of the center of mass of adsorbed drug, Rg is being used^36^. Calculated Rg values of the simulation systems are shown in Fig. 5. As shown, the Rg values for 3TC adsorbed on PEG are higher than adsorption on SiO_2_. Results confirm that the 3TC on the PEG complex is more stable and show that adsorption of 3TC on PEG lessens the energy levels and reaches more stability because the compactness of 3TC, when adsorbed on the PEG, is proved to be less. So, 3TC on PEG is wider and going to have more interactions.

### Radial distribution function (RDF)

**Figure 6:**
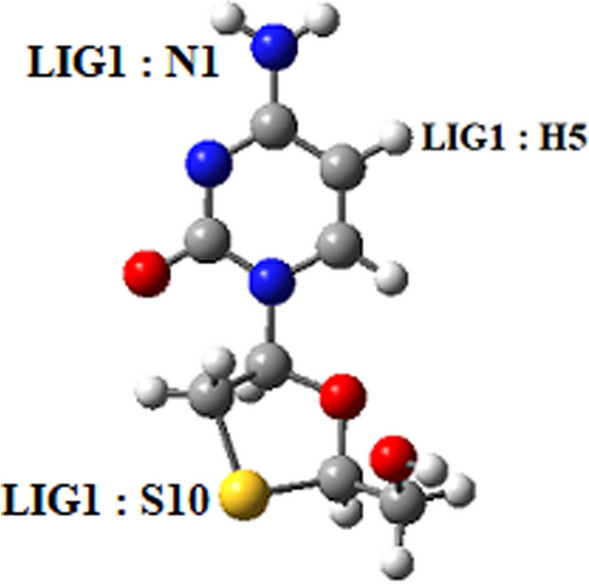
The position of H5, S10, and N1 atoms in 3TC molecule.

The orientation of the adsorbed drug molecules on both carriers, especially in systems with the possibility of hydrogen bond formation, plays an essential role in the system’s energy levels^9^. The RDF is an indicator that can be utilized to examine these accredits^39^. Figure 6 shows the position of the H5, S10, and N1 atoms in the 3TC molecule.

**Figure 7:**
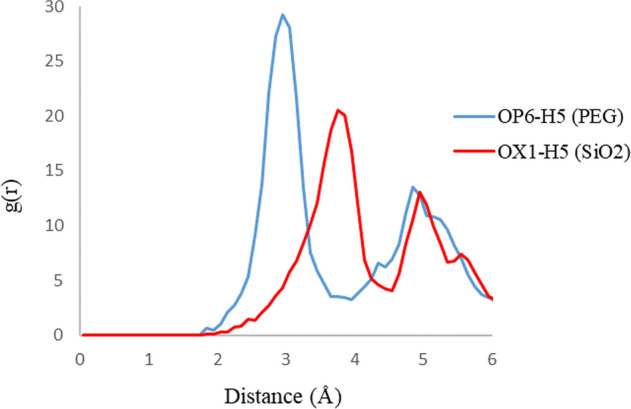
RDF plots for H5 atom of 3TC relative to the adsorption sites of SiO_2_ (red) and PEG (blue).

**Figure 8:**
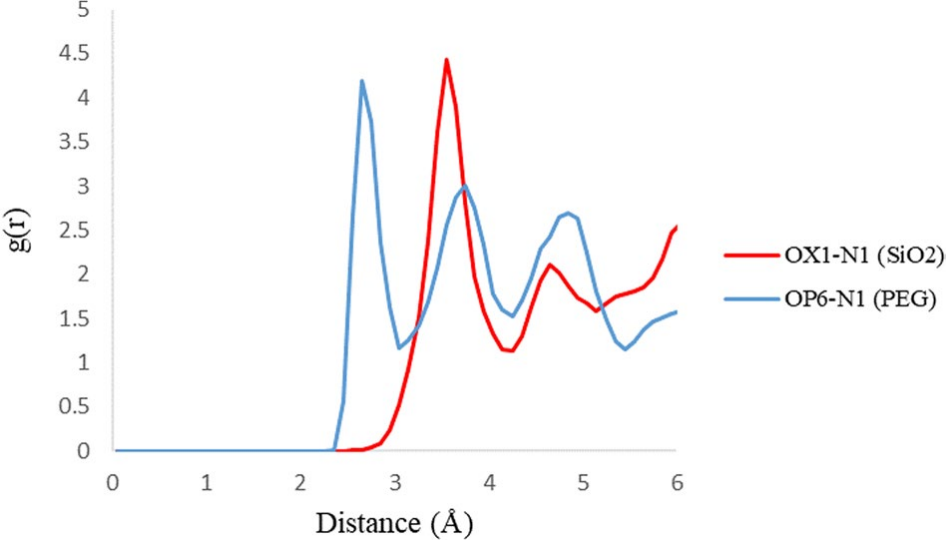
RDF plots for N1 atom of 3TC relative to the adsorption sites of SiO_2_ (red) and PEG (blue).

**Figure 9:**
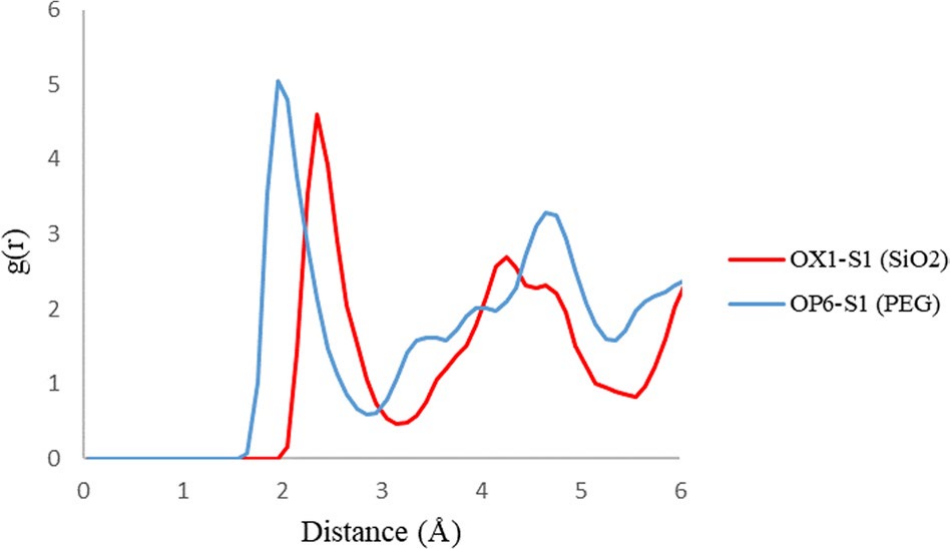
RDF plots for S1 atom of 3TC relative to the adsorption sites of SiO_2_ (red) and PEG (blue).

RDF plots are shown in Fig. 7 for the H5 atom of the 3TC molecule in adsorption with PEG and SiO_2_. The upper intensity of the peaks on PEG compared with SiO_2_ illustrates that 3TC on the PEG complex has more adsorption stability, and as it is shown, the PEG peak occurs at lower distances which can prove more drug loading. Figures 8 and 9 show RDF plots for S10 and N1 atoms relative to the adsorption sites in PEG (blue) and SiO_2_ (red) to check drug orientation in the adsorption process. It is observed that the drug is oriented nearly planer from the hexagonal ring site because of higher peaks of H5 of the ring rather than N1 and S1 atoms which proves the ring to be a planner and adsorbed to both carriers with main forces of hydrogen bonds.

### Hydrogen bonding

Hydrogen bonding involves a hydrogen atom placed between a pair of other atoms with a high affinity for electrons which is mainly weaker than a covalent bond but stronger than van der Waals forces^40^. The formation of hydrogen bonds is essential in the physical absorption process for drug delivery systems and the stability of drug structure on the nanocarrier^41^.

**Figure 10:**
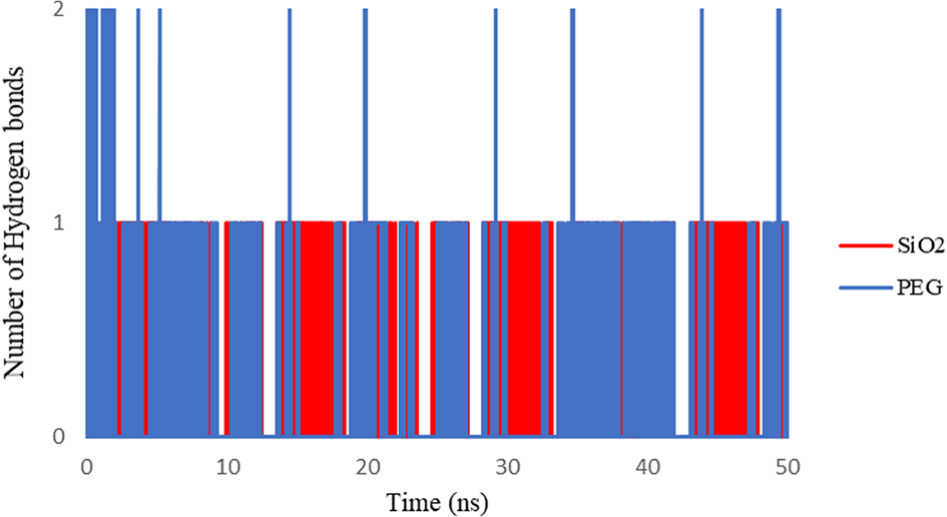
Number of hydrogen bonds between 3TC molecule and SiO_2_ (red) and PEG (blue) during the simulation time.

The number of hydrogen bonds between 3TC and carriers during the simulation time is computed. Figure 10 shows the number of hydrogen bonds as the most influential non-bonded interaction between 3TC on SiO_2_ and PEG. The results illustrate that the 3TC molecule adsorbed on PEG has more potential to form hydrogen bonds than SiO_2_. This feature leads to higher adsorption for 3TC on PEG, as confirmed by the RMSD results in Fig. 3.

### Non-bonded energies

**Figure 11:**
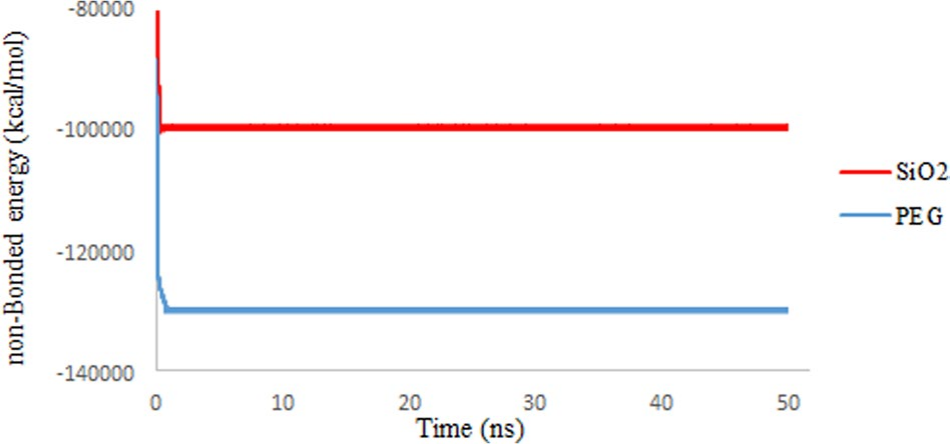
Non-bond interaction energies between 3TC and SiO_2_ (red), PEG (blue).

**Figure 12:**
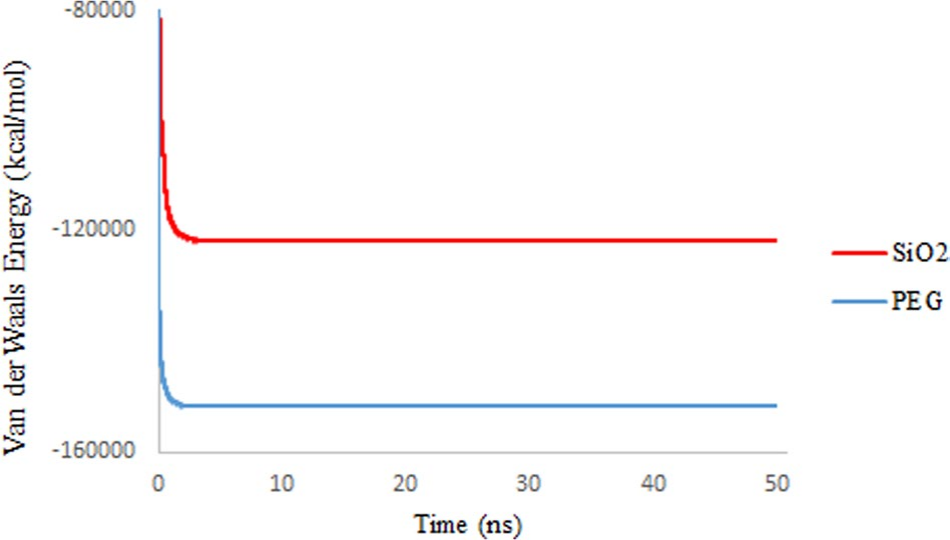
Van der Waals energies between 3TC and SiO_2_ (red), PEG (blue).

The amount of non-bonded energy in molecular systems is obtained by the algebraic sum of the coulomb and van der Waals (vdW) energies. The effect of vdW energy in this sum cannot be ignored even though the value is less. In general, the vdW energy varies mainly by two factors, surface area (molecular geometry) and electrical polarization capability (molecular size)^42^. Figures 11 and 12 show the total non-bonded energies and the share of vdW energies in simulation systems, respectively. The calculations show that the vdW energies are mainly related to the large size of the spherical carriers, which provide more place for electron distribution. In addition, the 3TC molecule shows fewer vdW interactions with SiO_2_ than PEG due to the more compact 3TC structure on SiO_2_, which is confirmed by Rg results. The results agree with the previous results that confirm absorption of 3TC on the PEG is more favorable in terms of energy.

### Drug loading

Since the main idea of using nanocarriers for antiviral drugs is to reduce the dosage a patient must take during his life, the concentration of drug molecules is a significant issue^43^. Therefore, the effect of drug concentration on the loading of 3TC on SiO_2_ and PEG is investigated. To this end, the drug concentration was increased one by one from 1 to 6 3TC molecules. Then 18, 24, and 48 3TC were added to fill the whole sphere in SiO_2_ and PEG. According to space barriers, there is no place for more drug molecules on carriers in the scale of our simulations.

**Figure 13:**
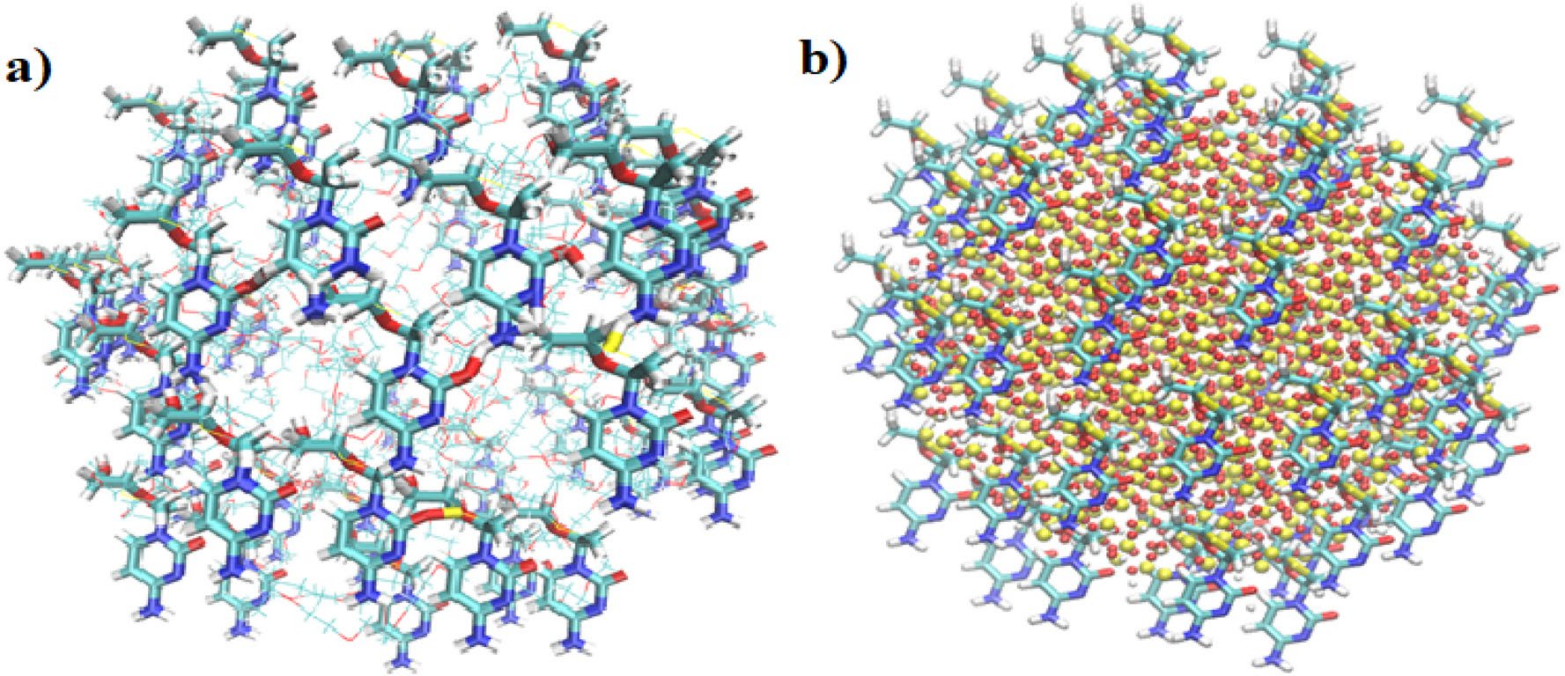
48 drug molecules on (**a**) PEG and (**b**) SiO_2_.

Figure 13 shows 48 3TC on each spherical carrier in the simulation scale. For more understanding of plots, 3, 6, 18, 24, and 48 3TC molecules were shown, while the 1, 2, 4, and 5 3TC molecules with the same procedure are neglected.

**Figure 14:**
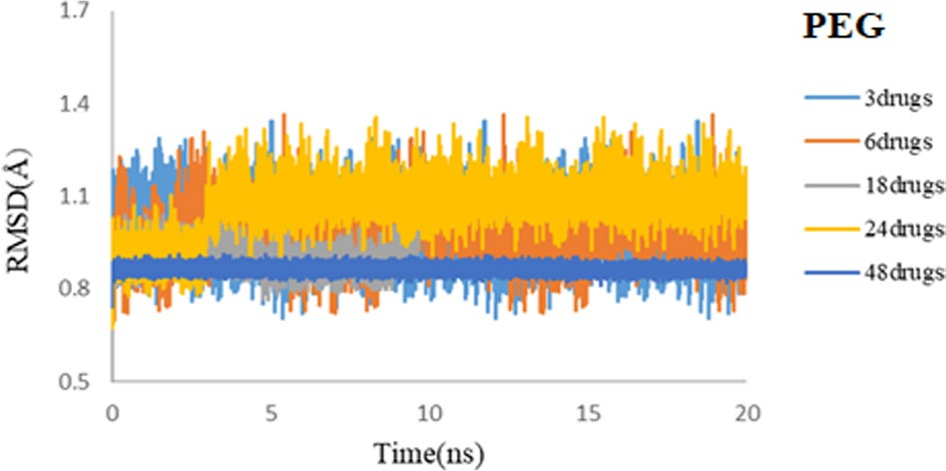
RMSD values of 3TC-PEG complex for 3, 6, 18, 24, and 48 drug molecules during 20 ns simulation time.

**Figure 15:**
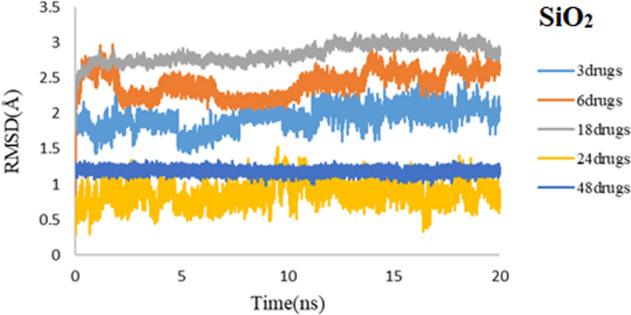
RMSD values of 3TC-SiO_2_ complex for 3, 6, 18, 24, and 48 drug molecules during 20 ns simulation time.

To determine the equilibrium of the studied systems, RMSD is computed, and the results are shown in Figs. 14 and 15 for PEG and SiO_2_, respectively. The RMSD plots reveal that all systems, either for SiO_2_ or PEG, have reached an equilibrium state in different drug concentrations. The stability of systems even with 48 3TC molecules that are shown in the coming plots illustrates that they are reliable nanocarriers for delivering high concentrations of antiviral drugs.

Convergence for all 3TC-PEG systems in RMSD studies is reached faster, showing less noise than the RMSD values for 3TC-SiO_2_. Results align with what we had discussed before that 3TC adsorbed on PEG illustrates more electrostatic interactions and provides more stable systems.

**Figure 16:**
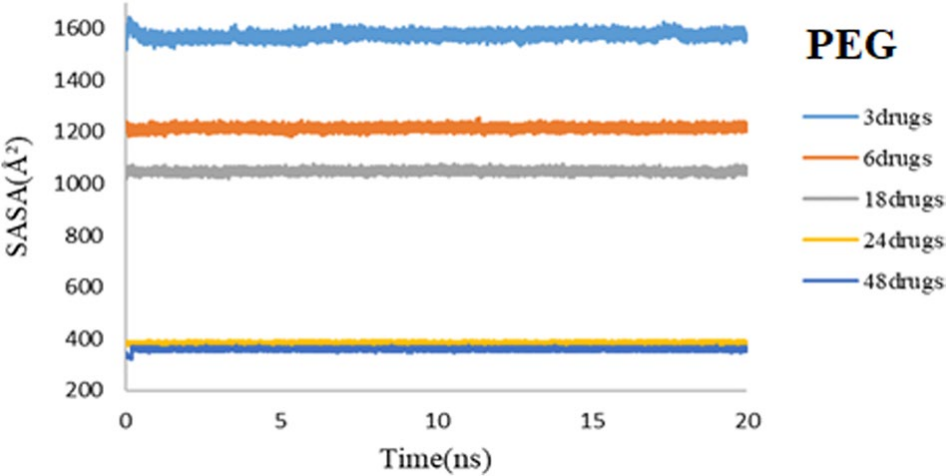
SASA values of 3TC-PEG complex for 3, 6, 18, 24, and 48 drug molecules during 20 ns simulation time.

**Figure 17:**
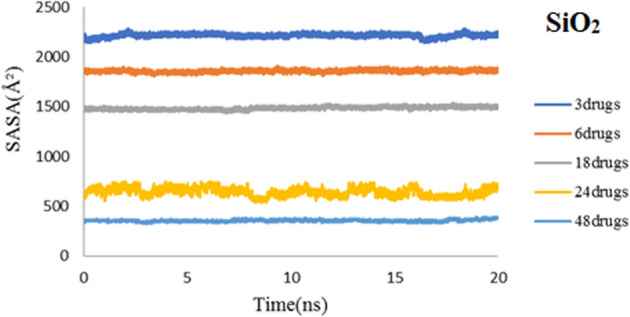
SASA values of 3TC-SiO_2_ complex for 3, 6, 18, 24, and 48 drug molecules during 20 ns simulation time.

As already noted, SASA is a crucial feature for determining the stability of drug-nanocarrier complexes. Figures 16 and 17 respectively show the SASA plots of PEG and SiO_2_. Adding to the concentration of drug molecules will reduce the surface area of the nanocarrier that is exposed to the solvent, which can be seen in both plots. In good agreement with previous data for one 3TC molecule, these results illustrate that the compactness of the 3TC-SiO_2_ complex in all studied systems is more than 3TC-PEG. SASA values for 3TC-PEG are converged to lower values rather than 3TC-SiO_2,_ proving that the adsorption of 3TC on PEG leads to more accumulated systems, and the 3TC-PEG complex is stuffed in higher concentrations of 3TC.

**Figure 18:**
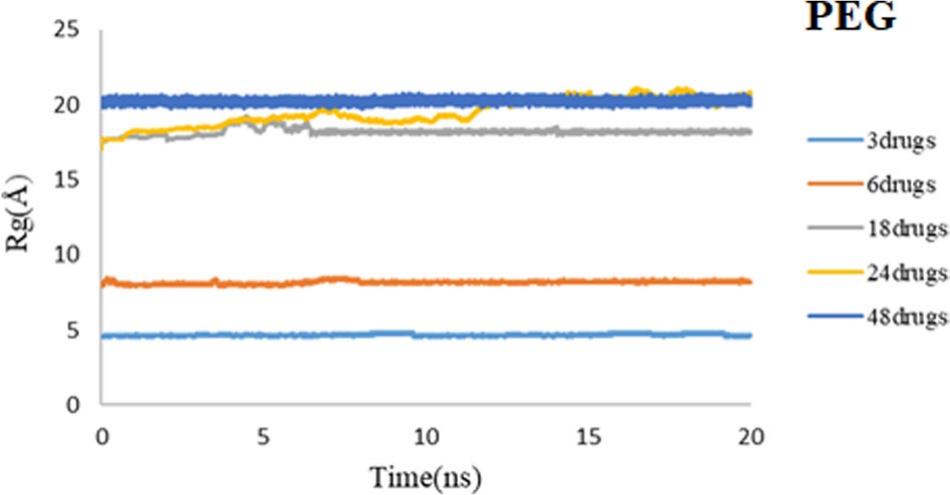
Rg values of 3TC adsorbed on PEG for 3, 6, 18, 24, and 48 drug molecules.

**Figure 19:**
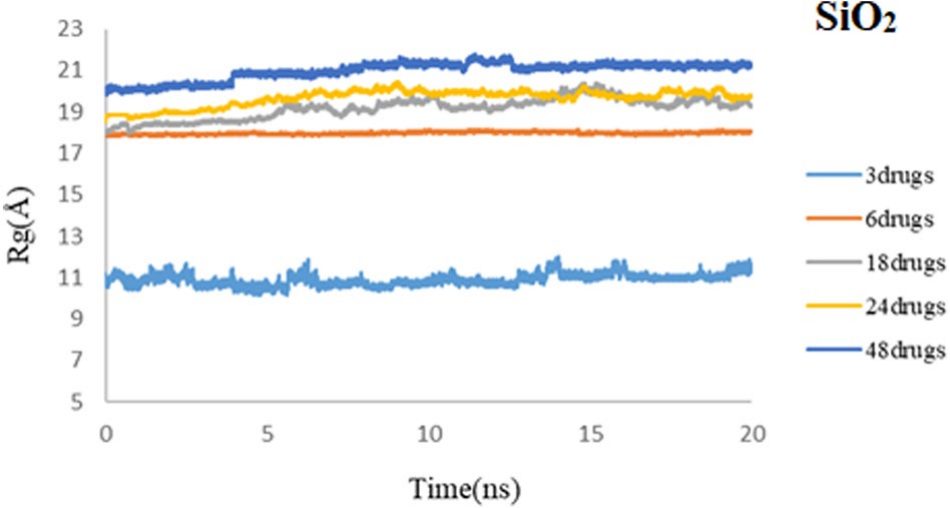
Rg values of 3TC adsorbed on SiO_2_ for 3, 6, 18, 24, and 48 drug molecules.

As discussed earlier, Rg mainly monitors the position of the center of mass of the drug and nanocarrier. Figures 18 and 19 represent the Rg results for all computed systems for PEG and SiO_2,_ respectively. Results demonstrate that in all five systems for both carriers, after 20 ns of simulation, they reached the equilibrium state. As mentioned before, it can be understood that using these spherical carriers may be reliable for high concentrations of 3TC molecules. It is shown that Rg values for 3TC-PEG are generally less than 3TC-SiO_2,_ which confirms previous results that have said the 3TC-PEG complex has more stability.

**Figure 20:**
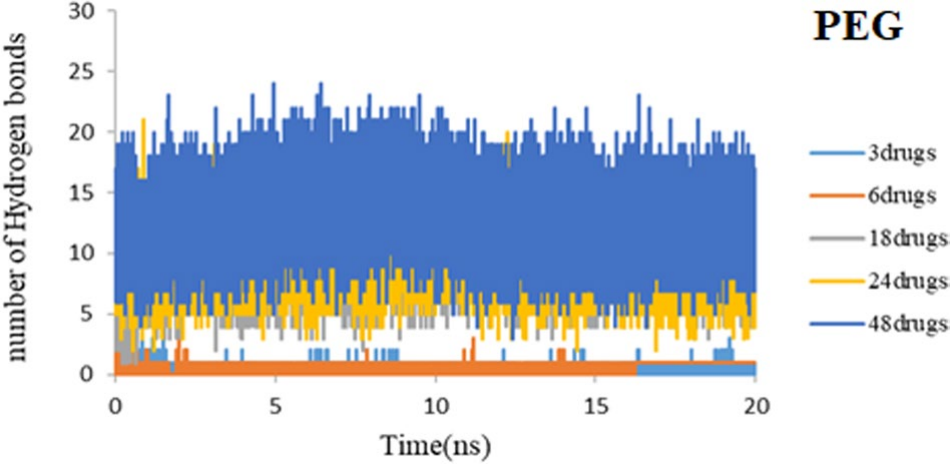
Number of hydrogen bonds between 3TC molecule and PEG during simulation time.

**Figure 21:**
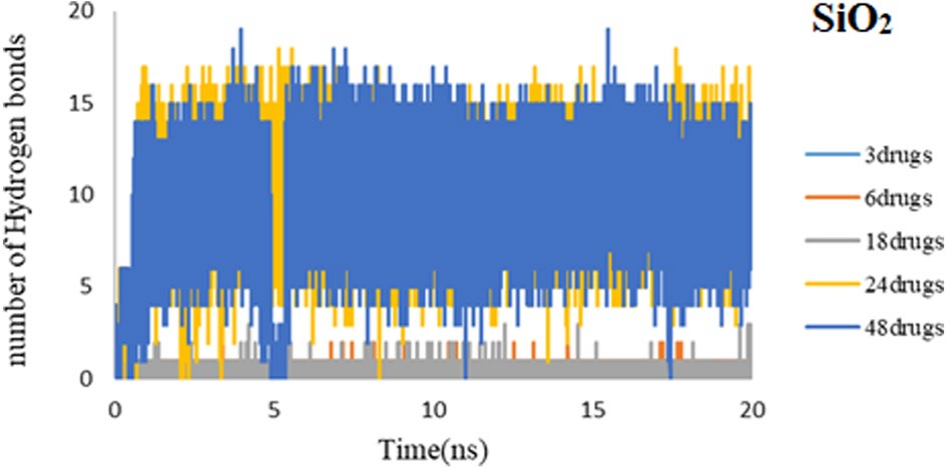
Number of hydrogen bonds between 3TC molecule and SiO_2_ during simulation time.

Hydrogen bonds append drugs to nanocarrier systems for drug delivery. The formation of hydrogen bonds plays a leading role in the stability of the drug-nanocarrier complex^35,36^. For all computed systems average number of hydrogen bonds per time is plotted during 20 ns of simulation time in Figs. 20 and 21. It can be seen that, in good agreement with previous results, the average number of H-bonds is more in each of the five systems in PEG rather than SiO_2_.

Molecular dynamics simulation is said to be an atomic scale laboratory. It provides a molecular modeling tool that can be used productively by a wide group of biomedical researchers, including particularly experimentalists. Having an insight into the adsorption process of 3TC on spherical PEG/SiO_2_ before the experiments can push scientists some steps forward in using nanocarriers to decrease the resistance of antiviral drugs. Our results on drug loading indicate excellent reliability for both carriers on high concentration of drugs. So they will be good candidates as nanocarriers to lessen the used drug dosage for patients every day. Notably, the previous experimental studies on SiO_2_ nanocarriers define it to be a well-founded agent for delivering antiviral drugs (double the drug effect)^7^. Also, this atomic view can help set the light on to utilize spherical nanocarriers for delivering antiviral drugs and trying to improve virus infected patient’s life. In this way experimental scientists have a brief atomic view of the structures and the adsorption process of the drug. So they can predict what will happen in the laboratory environment. In bio based studies this may lead to less use of animals and more accurate results^44^.

## Conclusion

In this study, MD simulation was utilized to carry out a parallel study on the adsorption process and loading of Lamivudine drug molecule on PEG and SiO_2_ as two high potential carriers for antiviral drugs. First, the adsorption of the 3TC molecule is simulated and analyzed on each carrier. The resulting data shows stronger adsorption of the 3TC on PEG than SiO_2_. The adsorption of the 3TC molecule on both carriers illustrates small RMSD fluctuations on average. Examinations of the 3TC molecule on the PEG/SiO_2_, including RMSD, Rg, SASA, RDF, and the number of hydrogen bonds, indicate that the adsorbed 3TC molecule on PEG has more stability. Increasing 3TC concentration, in the simulation scale, reveals the high loading capacity of PEG and also SiO_2_. In summary, this study verifies the former experimental studies in introducing spherical PEG/SiO_2_ as reliable carriers for drug delivery systems in body conditions and is a starting point in understanding the atomic view of drug delivery on spherical PEG and spherical SiO_2_ in aqueous solution.

## Data Availability

The datasets generated during the current study are available from the corresponding author on request.
